# Difference in default mode network subsystems in autism across childhood and adolescence

**DOI:** 10.1177/1362361320969258

**Published:** 2020-11-27

**Authors:** Joe Bathelt, Hilde M Geurts

**Affiliations:** 1 Royal Holloway, University of London, UK; 2 University of Amsterdam, The Netherlands

**Keywords:** autism spectrum disorders, brain development, default mode network, functional connectivity, modularity

## Abstract

Differences in the default mode network are among the most replicated brain-level findings in autistic individuals. Furthermore, subregions within the default mode network are associated with cognitive functions such as mentalising that are immediately relevant to cognitive theories of autism. Recent evidence suggests that the default mode network comprises partially independent subsystems that are tied to dissociable cognitive processes, specifically a medial temporal lobe subsystem involved in memory retrieval, a dorsal medial prefrontal cortex subsystem involved in social processing and a posterior cingulate cortex – anterior medial prefrontal cortex system that ties the other subsystems together. This modular organisation is thought to arise in childhood development. The current analysis investigated differences in default mode network subsystems in 193 autistic boys and young men (5–18 years) and in a group of 208 age-matched boys and young men without a diagnosis using resting-state functional magnetic resonance imaging from the data repository of the Autism Brain Imaging Data Exchange. The results indicated a developmental trend towards greater modularisation of the default mode network across childhood and adolescence in autism, mostly driven by reduced between-subnetwork connection strength. In contrast, default mode network subnetwork organisation was relatively stable in the comparison group. We suggest that these differences reflect delayed maturation of the default mode network in autism.

**Lay abstract:**

Neuroimaging research has identified a network of brain regions that are more active when we daydream compared to when we are engaged in a task. This network has been named the default mode network. Furthermore, differences in the default mode network are the most consistent findings in neuroimaging research in autism. Recent studies suggest that the default mode network is composed of subnetworks that are tied to different functions, namely memory and understanding others’ minds. In this study, we investigated if default mode network differences in autism are related to specific subnetworks of the default mode network and if these differences change across childhood and adolescence. Our results suggest that the subnetworks of the default mode network are less differentiated in autism in middle childhood compared to neurotypicals. By late adolescence, the default mode network subnetwork organisation was similar in the autistic and neurotypical groups. These findings provide a foundation for future studies to investigate if this developmental pattern relates to improvements in the integration of memory and social understanding as autistic children grow up.

## Introduction

Ever since non-invasive neuroimaging became available, research has tried to identify potential brain differences associated with autism. It became quickly apparent that no focal brain differences could be reliably identified across studies. Rather, the brains of autistic people seemed to be characterised by diffuse differences across large-scale brain systems comprising functionally coupled yet physically distant brain regions (Just et al., 2012; Kana et al., 2014). One such brain system that has been repeatedly implicated in autistic individuals is the default mode network (DMN; Padmanabhan et al., 2017). The DMN includes portions of the frontal cortex, the posterior midline and the inferior parietal lobule (Buckner & DiNicola, 2019). All DMN regions are part of the association cortex and show a protracted development that extends into the third decade of life (Buckner, 2012). The DMN was first identified in neurotypical participants as a network that showed reduced metabolic activity when participants where engaged in a task and, in contrast, became more active in the absence of task constraints (Binder et al., 1999; Gusnard et al., 2001). Further studies showed that DMN activity was not merely reflective of background metabolic activity, but was tied to specific cognitive processes. The current understanding suggests that the DMN is central for processing that requires internal representations, such as retrieval from autobiographical memory or self-referential reflection (Andrews-Hanna et al., 2010). Many regions of the DMN have been implicated in processes relevant to theories of autism, specifically the Theory of Mind (ToM) deficit theory (Baron-Cohen et al., 1989; Wing & Gould, 1979) and neural dysconnection syndrome theories (Frith, 2004; Geschwind & Levitt, 2007; Jones et al., 2009; Just et al., 2012; Kana et al., 2014). Regarding the reduced ToM theory, DMN regions, specifically the posterior cingulate cortex (PCC), medial prefrontal cortex (mPFC) and temporo-parietal junction (TPJ), have been implicated in self- and other-relevant processing, including in false belief and mentalising tasks (Carter et al., 2012; Castelli et al., 2002; Greicius et al., 2004; Kuzmanovic et al., 2011; Saxe & Kanwisher, 2003; Spreng et al., 2009). Studies in both autistic children and autistic adults reported reduced activity and/or connectivity in task-related and resting-state functional magnetic resonance imaging (fMRI) in these regions (Kana et al., 2015; Pantelis et al., 2015; von dem Hagen et al., 2013). Regarding dysconnection syndrome theories, the DMN contains some of the most highly connected and metabolically active regions across the whole brain (Leech & Sharp, 2013; Raichle et al., 2001). Any alterations to these hub regions are likely to lead to alterations in the global network architecture (van den Heuvel & Sporns, 2011).

Studies in recent years suggest that the DMN comprises three distinct yet closely connected subnetworks that serve dissociable cognitive processes (Andrews-Hanna et al., 2010, 2014; Buckner & DiNicola, 2019). One of the subnetworks is called the ‘medial temporal lobe (MTL) subsystem’ and includes the ventral medial prefrontal cortex (vMPFC), posterior inferior parietal lobule (pIPL), retrosplenial cortex (Rsp), parahippocampal cortex (PHC) and hippocampal formation (HF) (Andrews-Hanna et al., 2010). The MTL subsystem has been implicated in memory processes, such as episodic memory retrieval (Andrews-Hanna et al., 2014). Another subsystem termed the ‘dorsal medial prefrontal cortex (dMPFC) subsystem’ includes the dMPFC, temporo-parietal junction (TPJ), lateral temporal cortex (LTC) and temporal pole (TempP). This system has been implicated in mentalising and ToM (Andrews-Hanna et al., 2014). A third subsystem is made up of the anterior medial prefrontal cortex (aMPFC) and the PCC. This subnetwork forms a functional hub that ties the other DMN subsystems together (Andrews-Hanna et al., 2010) and is termed the ‘PCC-aMPFC core’.

The DMN plays a central role in brain theories of autism, but the subsystem composition and its development have only recently came to the fore. Different subsystems of the DMN may play particular role in the aetiology of autism given that some regions are directly tied to the core symptoms of autism, for example, the role of the TPJ for mentalising, while other regions may only be peripherally related to autism via a broader cognitive phenotype, for example, memory processes supported by the MTL subsystem. Two previous studies highlight the regional heterogeneity of the DMN in autism. Assaf and colleagues (2010) showed reduced connectivity within DMN subcomponents. In contrast, Lynch and colleagues (2013) reported increased connectivity between parietal and temporal regions in autistic children. Alterations in particular connections may have knock-on effects on the modular organisation of the DMN. Furthermore, shifts from general hyperconnectivity to hypoconnectivity in autism between childhood and adolescence (Uddin et al., 2013) may also affect the modular organisation of the DMN. Therefore, the current investigation aimed to investigate potential differences in DMN subsystem composition in autism. Moreover, we focused on age-related differences as the modular structure of the DMN has been suggested to arise between childhood and early adulthood (Buckner & DiNicola, 2019). We had three main expectations. First, we expected reduced connectivity for regions of the dMPFC subsystem based on the link between this subsystem and mentalising (Carter et al., 2012; Castelli et al., 2002; Greicius et al., 2004; Kuzmanovic et al., 2011; Saxe & Kanwisher, 2003; Spreng et al., 2009). Second, we expected higher connectivity based on reports of altered DMN organisation in autism (Cherkassky et al., 2006; Kennedy & Courchesne, 2008; Weng et al., 2009). Third, we expected a less modular organisation characterised by lower modularisation index (Rubinov & Sporns, 2011) and higher between-module connectivity in younger (i.e. children) compared to older (i.e. adolescents) participants (Buckner & DiNicola, 2019).

## Materials and methods

### Participants

The current analysis made use of publicly available data from the Autism Brain Imaging Data Exchange (ABIDE, Martino et al., 2014; ABIDE-II, Martino et al., 2017). Both the ABIDE and ABIDE-II database contain data from participants with a diagnosis of autism spectrum condition (abbreviated as ASC from here on) and a comparison group of individuals without a diagnosis (abbreviated as CMP from here on).^1^ We only included male participants under 18 years of age to reduce the variability in the sample (Alaerts et al., 2016; Kozhemiako et al., 2020; Lawrence et al., 2020). Furthermore, we did not include acquisition sites with an eyes-open resting-state protocol to keep the acquisition consistent across the included sample. The initial sample after applying these criteria comprised 299 participants in the ASC group and 266 in the CMP group. To mimise the influence of low-quality scans, we excluded scans that were rated as low quality by human experts (*n* = 88) or fell outside of the recommend range on image quality metrics (frame-wise displacement (FD) > 0.5 mm: *n* = 46; DVARS > 5%: *n* = 24). Furthermore, we excluded participants at the extreme of the distribution for full-scale IQ (<5%ile (standard score < 83): *n* = 27; >95%ile (standard score > 130): *n* = 21). The final sample comprised 401 participants (ASC: *n* = 193, CMP: *n* = 208; see Table 1 for sample characteristics) from 14 acquisition sites. To investigate the association between symptom scores and DMN structure, we focused on the Social Responsiveness Scale total score (SRS, *z*-scaled raw scores) and the ADOS-G total scores (*z*-scaled raw score) because these measures were available for the largest sub-sample of participants in the ASC group (SRS: *n* = 102, age: 11.31 ± 3.045; ADOS: *n* = 94, age: 12.58 ± 2.803 (mean ± SD)).

**Table 1.** Overview of sample characteristics (ASC: *n* = 193; CMP: *n* = 208).

Mean	SD	Min	Max	
Age (years)				
ASC	12.34	3.013	5.53	18.00
CMP	12.66	2.282	6.36	18.00
FIQ (scaled score)				
ASC	107.04	11.669	84	129.50
CMP	110.36	10.639	84	129.00
FD (mm)				
ASC	0.18	0.098	0.03	0.49
CMP	0.15	0.090	0.04	0.49
DVARS (%)				
ASC	3.36	0.705	1.35	4.73
CMP	2.87	0.678	1.36	4.55

ASC: autism spectrum condition; CMP: comparison; SD: standard deviation; FIQ: Full-Scale IQ; FD: frame-wise displacement (according to Power et al. (2014)); DVARS: derivative of the variance.

Please note that only male participants were included in the sample for this analysis.

### fMRI processing

#### Preprocessing

The current analysis was based on preprocessed data made available by the Preprocessed Connectome Project to ensure the replicability of the findings (http://fcon_1000.projects.nitrc.org/indi/abide/). We used data processed using fMRIPrep, a state-of-the-art fMRI processing pipeline *fMRIPrep* v1.2.5 (PRID:SCR_016216). To briefly summarise the processing step, the T1-weighted (T1w) image was corrected for intensity non-uniformity (INU) using *N4BiasFieldCorrection* (ANTs 2.2.0) and used as T1w-reference throughout the workflow. The T1w-reference was then skull-stripped using *antsBrainExtraction.sh* (ANTs 2.2.0), using OASIS as the target template. Brain surfaces were reconstructed using *recon-all* (Dale et al., 1999; FreeSurfer 6.0.1, RRID:SCR_001847), and the brain mask estimated previously was refined with a custom variation of the method to reconcile ANTs-derived and FreeSurfer-derived segmentation of the cortical grey-matter of Mindboggle (Klein et al., 2017; RRID:SCR_002438). Spatial normalisation to the ICBM 152 Nonlinear Asymmetrical template version 2009c (Fonov et al., 2009; RRID:SCR_008796; see ‘Considerations regarding standard space transformation’ in the Supplementary Materials) was performed through nonlinear registration with *antsRegistration* (Avants et al., 2007; ANTs 2.2.0, RRID:SCR_004757), using brain-extracted versions of both T1w volume and template. Brain tissue segmentation of cerebrospinal fluid (CSF), white matter (WM) and grey matter (GM) was performed on the brain-extracted T1w using *FAST* (Zhang et al., 2001; FSL 5.0.9, RRID:SCR_002823).

For each BOLD run per subject, the following preprocessing was performed. First, a reference volume and its skull-stripped version were generated using a custom methodology of *fMRIPrep*. The BOLD reference was then co-registered to the T1w reference using *bbregister* (FreeSurfer) which implements boundary-based registration (Greve & Fischl, 2009). Co-registration was configured with 9 degrees of freedom to account for distortions remaining in the BOLD reference. Head-motion parameters with respect to the BOLD reference (transformation matrices, and six corresponding rotation and translation parameters) are estimated before any spatiotemporal filtering using *mcflirt* (Jenkinson et al., 2002; FSL v5.0.9). The BOLD time series were resampled onto their original, native space by applying a single, composite transform to correct for head-motion and susceptibility distortions. The BOLD time series were resampled to MNI152NLin2009cAsym standard space, generating a preprocessed BOLD run in MNI152NLin2009cAsym space. Several confounding time series were calculated based on the preprocessed BOLD: FD, DVARS and three region-wise global signals. FD and DVARS were calculated for each functional run, both using their implementations in *Nipype* (following the definitions by Power et al, 2014). Global signals were extracted within a CSF and a WM mask defined in the anatomical image for confound regression.

#### Calculation of DMN connectivity

Prior to time-series extraction, the effect of 32 nuisance signals (CSF, WM, rotation and translation in three directions, their temporal derivative, squared temporal derivative and squared term, see Satterthwaite et al., 2012) and the first five principal components of the 5% most variable voxels (*CompCor*; Behzadi et al., 2007) was regressed from each voxel. Furthermore, the data were filtered using a band-pass filter (0.008–0.1 Hz), spatially smoothed (6 mm FWHM) and de-trended using nilearn v0.5.2 (Abraham et al., 2014; RRID:SCR_001362). Subsequently, the time series for regions of interest (ROIs) within the DMN was extracted by averaging the signal within spheres (radius: 8 mm) centred at the DMN coordinates from a seminal study on DMN subnetworks (Andrews-Hanna et al., 2010; see Table 1, see ‘Considerations regarding ROI defintion’ in the Supplementary Materials). Next, the same regressors that were used to correct nuisance signals at the voxel level were regressed from the ROI time series (Abraham et al., 2017) and the correlation between the time series residuals of all pairwise combinations of ROIs was calculated. For statistical analysis, correlations were transformed using Fisher’s *r*-to-*z* transform.

The data quality may differ between ROIs, which could confound the connectivity analysis. To investigate differences in signal quality between the ROIs, we calculated the BOLD SNR according to the definition by Kruger and Glover (2001) as implemented in MRIQC v0.15.1. There was no significant difference in SNR between ROIs (one-way analysis of variance (ANOVA): *F*(10, 3278) = 0.79, *p* = 0.640). We also checked if the interaction between ROI and acquisition site was associated with SNR to account for differences in SNR between acquisition sites but did not find a significant interaction (ANOVA: site × ROI: *F*(110, 3157) = 0.529, *p* > 0.999).

**Figure 1.**
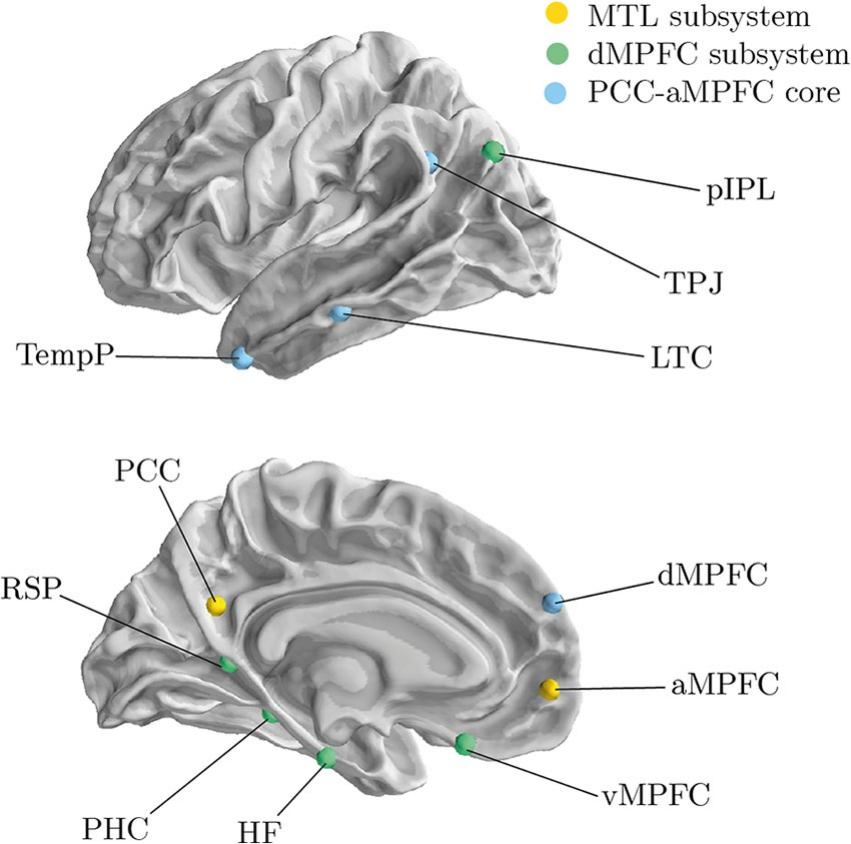
Illustration of DMN ROIs. The colour indicates the subsystem associated with each node. aMPFC: anterior medial prefrontal cortex; dMPFC: dorsal medial prefrontal cortex; HF: hippocampal formation; LTC: lateral temporal cortex; MTL: medial temporal lobe; PCC: posterior cingulate cortex; PHC: parahippocampal cortex; pIPL: posterior inferior parietal lobe; RSP: retrosplenial cortex; TempP: temporal pole; TPJ: temporal parietal junction; vMPFC: ventral medial prefrontal cortex.

To characterise the structure of the DMN subdivisions, we focused on a network measure called modularity. Within network science, modularity is the degree to which a network can be divided into separate modules that show higher connectivity between the nodes within that module than with the rest of the network. Modularity can be characterised through the modularity index which is based on the ratio of within- versus between-module connections for a given module division of the network. Higher modularity index values indicate a greater modular organisation of the network. The modularity index was calculated based on the weighted, undirected connectivity matrices as described by Rubinov and Sporns (2011). The module solution identified by Andrews-Hanna et al. (2010) was used for this purpose (Figure 1 and Table 2).

**Table 2.** Labels, coordinates and subsystem membership of DMN ROIs in the current analysis taken from Andrews-Hanna et al. (2010).

Region	*x*	*x*	*z*	Subsystem
Ventral medial prefrontal (vMPFC)	0	26	−18	MTL Subsystem
Posterior inferior parietal lobe (plPL)	−44	−74	32	MTL Subsystem
Retrosplenial cortex (Rsp)	−14	−52	8	MTL Subsystem
Parahippocampal cortex (PHC)	−28	−40	−12	MTL Subsystem
Hippocampal formation (HF)	−22	−20	−26	MTL Subsystem
Dorsal medial prefrontal cortex (dMPFC)	0	52	26	dMPFC Subsystem
Temporal parietal junction (TPJ)	−54	−54	28	dMPFC Subsystem
Lateral temporal cortex (LTC)	−60	−24	−18	dMPFC Subsystem
Temporal pole (TemP)	−50	14	−40	dMPFC Subsystem
Anterior medial prefrontal cortex (aMPFC)	−6	52	−2	PCC-aMPFC Core
Posterior cingulate cortex (PCC)	−8	−56	26	PCC-aMPFC Core

DMN: default mode network; ROIs: regions of interest; vMPFC: ventral medial prefrontal cortex; MTL: medial temporal lobe; plPL: posterior inferior parietal lobe; Rsp: retrosplenial cortex; PHC: parahippocampal cortex; HF: hippocampal formation; dMPFC: dorsal medial prefrontal cortex; TPJ: temporo-parietal junction; LTC: lateral temporal cortex; TemP: temporal pole; aMPFC: anterior medial prefrontal cortex; PCC: posterior cingulate cortex.

#### Statistical analysis

To account for non-normality and large variability in the data, we performed permutation-based statistical analyses. For this purpose, we created 1000 bootstrap samples of the original data and of data with scrambled outcome-predictor associations. In each bootstrap sample, we fitted a general linear model (GLM) with predictors of age (continuous), group (ASC, CMP) and their interaction (age × group). Furthermore, the model contained full-scale IQ as a nuisance regressor. Please note that we did not include acquisition site as a nuisance regressor, because site was confounded with age (one-way ANOVA: *F*(19, 381) = 17.24, *p* < 0.001, $\eta^{2}=0.46$, $\omega^{2}=0.43$). Instead, we accounted for between-site differences in image quality. We obtained *p* values by comparing the bootstrapped median regression coefficient to the distribution of coefficients obtained with the scrambled data. We applied false discovery rate (FDR) correction using the Benjamini–Hochberg method to account for multiple comparisons across ROIs. For comparisons of summed connection strengths, we applied Bonferroni correction. All reported *p* values are corrected for multiple comparisons. While age was treated as a continuous variable for all statistical analyses, we split the ASC and CMP group into an older and a younger group for visualisation purposes using median splits in each group (ASC younger: *n* = 96, age: mean = 9.89, SD = 1.705; ASC older: *n* = 96, age: mean = 14.81, SD = 1.759; CMP younger: *n* = 104, age: mean = 10.23, SD = 1.459; CMP older: *n* = 104, age: mean = 15.09, SD = 1.599).

To investigate the association between autism symptoms and DMN connectivity, we used separate GLMs with symptom score as the outcome and DMN measures as the predictor (modularity index, between-module connection strength, MTL module connection strength, dMPFC module connection strength, core module connection strength). We also included full-scale IQ, the four image quality factors (see ‘Results’ for a description) and age (linear, squared) as predictors of no interest. We applied the same bootstrap resampling method as described above to evaluate the model.

The code to reproduce the analyses is available on the Open Science Framework website (https://osf.io/y835k/?view_only=bdedc67a1a044589918ad6b528353e6b).

## Results

The number of participants per acquisition site did not differ between the groups (ASC compared to average: $\chi^{2}=17.17$, *p* = 0.578; CMP compared to average: $\chi^{2}=17.39$, *p* = 0.564). The groups showed no significant difference in age (Welch-corrected *t* test: *t*(393.12) = −1.08, *p* = 0.279, *d* = −0.11). There was a difference in full-scale IQ scores with slightly lower scores in the ASC group (*t*(388.23) = −2.97, *p* = 0.001, *d* = 0.34). There was also a difference in image quality as indicated by more FD (*t*(389.44) = 3.4, *p* = 0.001, *d* = 0.34) and higher DVARS (*t*(393.62) = 2.35, *p* = 0.019, *d* = 0.24) in the ASC group. In addition, there was a higher number of movement outliers in the ASC group, defined as a movement score (FD or DVARS) above the 95th percentile across groups (FD: ASC: *n* = 11; CMP: *n* = 9, $\chi^{2}=1.90$, *p* = 0.168; DVARS: ASC: *n* = 13, CMP: *n* = 7, $\chi^{2}=1.90=7.34$, *p* = 0.007). To account for differences in image quality, we ran a principal component analysis on the 30 image quality metrics generated by MRIQC v0.14 (see Supplementary Materials for full list). A four-factor solution provided the best account of the image quality data (see Supplementary Materials for details). To check that the image quality factors removed between-site confounds, we compared the performance of support vector machine (SVM) classification tasked with predicting acquisition site from the DMN connectivity values. We trained the SVM models in a random selection of two-thirds of the data and tested the performance in the held-out third. Using the native connectivity values, the accuracy of the prediction in unseen data was 0.26 (accuracy adjusted for unequal class sizes). When regressing the quality values, the accuracy dropped to 0.16. In contrast, when using ‘site’ as a categorical regressor, the accuracy was 0.17.

**Figure 2.**
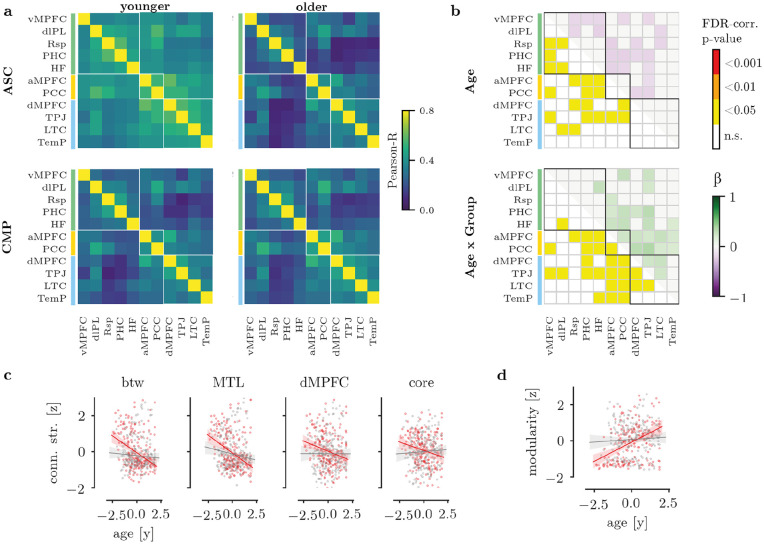
Differences in DMN connections. (a) Average adjacency matrix in younger (left) and older (right) participants in the ASC (top) or CMP (bottom) group. White boxes indicate the boundaries of subnetworks (green: MTL subsystem; yellow: PCC-aMPFC core; blue: dMPFC subsystem). Please note that age was treated as a continuous variable in the main analysis. The age split is only shown for illustration purposes. (b) Results of the statistical analysis. The upper matrix shows significant age effects with FDR-corrected *p* values in the lower triangle and the standardised regression coefficient (β) in the upper triangle. The lower matrix shows the age × group interaction. (c) Regression results for the association between age and connection strength in the ASC (red) and CMP (grey) group per region. The regression lines and confidence intervals were based on 5000 bootstrap samples drawn from the original data. (d) Regression results for the association between age and modularity in the ASC (red) and CMP (grey) group. btw: between module; MTL: MTL subsystem; dMPFC: dMPFC subsystem; core: PCC-aMPFC subsystem.

The analysis of connection strengths indicated significantly lower connection strength in the older compared to younger participants, particularly for connections between subsystems (see Figure 2(a) and (b)). The association between age and connection strength was stronger in the ASC compared to the CMP group as indicated by significant age × group interactions. The largest interaction effects were observed for between-module connections (see Figure 2(b)).

The analysis of summed connection strength between subsystems indicated a significant age × group interaction with greater reduction in connection strength in the ASC group (age: *p* < 0.001, group: *p* = 0.656, age × group: *p* = 0.012; see Figure 2(c)). This interaction was also observed when excluding motion outliers (age: *p* < 0.001, group: *p* = 0.768, age × group: *p* = 0.048). The connection strength of the MTL subsystem was lower in older participants in both groups (age: *p* = 0.004, group: *p* > 0.999, age × group: *p* = 0.064; see Figure 2(c)). This effect was also observed when excluding motion outliers (age: *p* < 0.001, group: *p* > 0.999, age × group: *p* = 0.128). The dMPFC subsystem showed a significant association of age with connection strength (dMPFC: age: *p* = 0.028, group: *p* > 0.999, age × group: *p* = 0.072; see Figure 2(c)). The age effect in the dMPFC subsystem remained when excluding motion outliers (age: *p* = 0.028, group: *p* = 0.508, age × group: *p* = 0.276). There were no significant age or age × group effects for the core system (age: *p* = 0.052, group: *p* = 0.996, age × group: *p* = 0.044). There was no significant association between connection strength in any module and SRS scores (*n* = 102, between: β=0.06 (−0.104, 0.215), *p* > 0.999; MTL: β=0.06 (−0.094, 0.213), *p* > 0.999; core: β=0.12 (−0.296, 0.039), *p* > 0.999; dMPFC: β=0.05 (−0.132, 0.231), *p* > 0.999; median (5%ile, 95%ile) based on 1000 bootstrap permutations). There was also no association between connection strength and ADOS scores (*n* = 94; between: β=0.15 (−0.028, 0.342), *p* = 0.292; MTL: β=0.16 (−0.010, 0.339), *p* = 0.236; core: β=−0.15 (−0.334, 0.042), *p* > 0.999; dMPFC: β=0.19 (0.0049, 0.351), *p* = 0.136).

The modularity index show a significant age × group interaction with a lower modularity in younger participants with ASC but similar modularity in the ASC and CMP group at older ages (age: *p* = 0.001, group: *p* = 0.086, age × group: *p* = 0.001; see Figure 2(d)). The age and interaction effects remained when excluding motion outliers (age: *p* = 0.001, group: *p* = 0.122, age × group: *p* = 0.007). There was no significant association between the modularity index and SRS scores or ADOS scores (SRS: *n* = 102, β=−0.12 (−0.288, 0.057), *p* = 0.889; ADOS: *n* = 94, β=−0.14 (−0.324, 0.046), *p* = 0.921).

## Discussion

This study investigated differences in DMN subnetworks in autism across childhood and adolescence. The results indicated more pronounced decreases in connection strength in the ASC group, particularly for connections between DMN subnetworks (Hypothesis 1). Also contrary to our expectation, the differences in the ASC group were tied to differences in between-subnetwork connection strength rather than differences in the dMPFC subsystem (Hypothesis 2). In line with our expectation (Hypothesis 3), the results indicated a less modular organisation as indicated by more between-subnetwork connections and a lower modularisation index in autism, but mostly in younger participants.

In line with previous studies, the results of the current analysis indicated higher connectivity within the DMN in younger autistic children (Glerean et al., 2015; Lynch et al., 2013; Moseley et al., 2015). This difference was attenuated in adolescents. The strongest decreases in connection strength were observed in between-subnetwork connections, which was also reflected in a higher modularity index with age. Previous studies in a similar age range provided inconsistent results with some studies reporting higher connectivity and others reporting lower connectivity in the DMN (Assaf et al., 2010; Cheng et al., 2015; Jung et al., 2014; Monk et al., 2009; Supekar et al., 2013; Washington et al., 2013; Weng et al., 2009). These inconsistencies have been attributed to differences in the age composition of samples. Uddin and colleagues (2013) suggested that functional connectivity differences in ASC shift from relative hyperconnectivity in early development towards relative hypoconnectivity in adolescence and adulthood. Consistent with this suggestion, this study indicated higher DMN subnetwork connectivity in childhood in autism that shifts towards lower connectivity and greater DMN modularity in adolescence. There was no indication for DMN hypoconnectivity in adolescence, instead DMN subnetwork connection strength appeared similar in the ASC and neurotypical groups towards the end of the included age range (18 years). Potential hypoconnectivity as suggested by Uddin and colleagues may arise in later development (>18 years). Alternatively, the reduction in hyperconnectivity in the ASC group may suggest a delayed developmental pattern. A recent authoritative review on the DMN (Buckner & DiNicola, 2019) suggests that the modularisation of the DMN subsystems is an activity-dependent developmental process. According to this model, the proto-DMN is less specialised and more distributed in early typical development. Activity within the MTL subsystem is driving the subsequent specialisation towards the MTL and dMPFC subsystems that serve specific processes. The results of the current analysis of greater between-subnetwork connectivity and age-by-group differences within the MTL subsystem may suggest that the development of DMN subsystem modularisation may unfold differently in autism. However, a younger age range in the neurotypical group would be required to investigate the developmental delay hypothesis.

There are some limitations to this study. Between-site variability is the most challenging limitation of this study. As age and site were confounded, we could not follow the analyses which are typically run with the ABIDE data sets. However, our approach to account for signal quality removed some of the differences between sites. Yet, in addition to variation in technical aspects, the samples from different sites likely vary in other respects. Autism is extremely variable with many factors that may introduce variation such as pharmacological or behavioural intervention, age of onset, overall disability and symptom profile that could not be controlled in the current analysis. Large data collection efforts that acquire neuroimaging and phenotypic data from multiple sites with a harmonised protocol will be needed to account for this heterogeneity, such as the ongoing *European Autism Interventions – A Multicentre Study for Developing New Medications* (EU AIMS) initiative. Second, to reach a sufficient number of participants, we had to rely on cross-sectional data, which limits the conclusions that can be drawn about developmental trajectories. Future longitudinal studies are needed to investigate individual trajectories to firmly establish the development of the DMN subnetworks in autism. Second, the current analysis was based on data collated from several studies with no harmonisation of the acquisition protocol, which may introduce confounds. Third, this study focussed on autistic males. Sex has been found to influence connectivity, including connectivity of the DMN (Floris et al., 2018; Ypma et al., 2016). Differences in DMN development in autistic females remain to be investigated in future research. Fourth, the available phenotypic characterisation of the ASC group was limited and only available for half of the autism group. Future dedicated studies that combine out-of-scanner behavioural testing and potentially task fMRI assessments will be best suited to establish these associations.

In summary, the current analysis investigated differences in DMN subsystem organisation in autism across childhood and adolescence. The results indicated a developmental trend towards greater modularisation of the DMN, mostly driven by a reduction in between-subnetwork connectivity. We suggest that this may reflect a delayed maturation of the DMN in autistic males. These results may inform future studies on the brain-level aetiology of autism across development.

## Supplemental Material

sj-pdf-1-aut-10.1177_1362361320969258 – Supplemental material for Difference in default mode network subsystems in autism across childhood and adolescence Click here for additional data file. Supplemental material, sj-pdf-1-aut-10.1177_1362361320969258 for Difference in default mode network subsystems in autism across childhood and adolescence by Joe Bathelt and Hilde M Geurts in Autism

## Notes

1. A note on nomenclature: We use the term of ‘autism spectrum condition’ throughout this article because the traditionally used term ‘autism spectrum disorder’ carries unjustified negative connotations. With regard to the label ‘comparison group’, we were aiming for the most accurate description of the group of participants without a diagnosis. The term ‘comparison group’ is used because the term ‘control group’ is reserved for experimental settings in which the researcher has control over the group assignment. The analysis presented in the article was based on groups that were recruited based on their diagnosis and were not randomly assigned. Therefore, it would not be accurate to use the term ‘control’ to describe the group without a diagnosis. Furthermore, it was not assessed if the group without a diagnosis was in fact ‘typically developing’ or ‘neurotypical’. For that reason, we prefer to not apply these labels.
