# Supplementary material for: Difference in default mode network subsystems in autism across childhood and adolescence
Source: Autism. 2020 Nov 27;25(2):556–65. doi: 10.1177/1362361320969258 (PMC7874372; doi:10.1177/1362361320969258)
Supplement: sj-pdf-1-aut-10.1177_1362361320969258 – Supplemental material for Difference in default mode network subsystems in autism across childhood and adolescence [file sj-pdf-1-aut-10.1177_1362361320969258.pdf]

Supplementary Materials

Scan Protocols

The final sample comprised participant data from 14 acquisition sites. The protocol differed between the sites. The table below summarises the BOLD fMRI and T1w protocol for each site.

Table S1: *Scanning protocol of all acquisition sites that were included in the analysis. Site abbreviations according to the nomenclature of ABIDE and ABIDE-II. See* http://preprocessed-connectomes-project.org *for details.*

ABIDE

Site	KKI	Leuven_1	Max_Mun	NYU	OHSU
number included	20	2	5	71	16
Manufacturer	Philips	Philips	Siemens	Siemens	Siemens
Model	Achieva	Intera	Verio	Allegra	TrioTim
Field Strength [T]	3	3	3	3	3
BOLD fMRI					
Echo Time [s]	0.03	0.033	0.03	0.015	0.03
Repetition Time [s]	3	3	3	3	3
Pixel spacing [mm]	3.05x3.15	3.59x3.59	3x3	3x3	3.8x3.8
Slice thickness [mm]	3	4	3	4	3.8
Orientation	j-	j-	j-	i	j-
Flip Angle [deg]	75	90	80	90	90
Duration [min]	06:40	07:06	10:06	06:00	03:32
T1					
Repetition Time [s]	3.5	3	1.8	2.53	2.3
Echo Time [s]	0.0037	0.0046	0.00306	0.00325	0.003589
Flip Angle [deg]	8	8	9	7	10

Site	Olin	SDSU	UCLA_1	UCLA_2	UM_1
number included	11	19	49	16	46
Manufacturer	Siemens	GE	Siemens	Siemens	GE
Model	Allegra	MR750	TrioTim	TrioTim	Signa
Field Strength [T]	3	3	3	3	3
BOLD fMRI					
Echo Time [s]	0.027	0.03	0.028	0.028	0.03
Repetition Time [s]	3	3	3	3	3
Pixel spacing [mm]	3.4x3.4	3.4x3.4	3x3	3x3	3.438x3.438
Slice thickness [mm]	4	3.4	4	4	3
Orientation	j-	j-	j-	j-	j-
Flip Angle [deg]	60	90	90	90	90
Duration [min]	05:15	06:10	06:06	06:06	10:00
T1					
Repetition Time [s]	2.5	0.01108	2.3	2.3	N/A

Echo Time [s]	0.00274	0.0043	0.00284	0.00284	0.0018
Flip Angle [deg]	8	8	9	9	15

Site	UM_2	Yale
number included	27	17
Manufacturer	GE	Siemens
Model	Signa	TrioTim
Field Strength [T]	3	3
BOLD fMRI		
Echo Time [s]	0.03	0.025
Repetition Time [s]	3	3
Pixel spacing [mm]	3.438x3.438	3.4x3.4
Slice thickness [mm]	3	4
Orientation	j-	j-
Flip Angle [deg]	90	60
Duration [min]	10:00	06:40
T1		
Repetition Time [s]	N/A	1.23
Echo Time [s]	0.0018	0.00173
Flip Angle [deg]	15	9

ABIDE-II

Site	ETH_1	GU_1	KUL_3	NYU_1	NYU_2
number included	2	28	4	14	11
Manufacturer	Philips	Siemens	Philips	Siemens	Siemens
Model	Achieva	TriTim	Achieva Ds	Allegra	Allegra
Field Strength [T]		3	3	3	3
BOLD fMRI					
Echo Time [s]	0.025	0.03	0.03	0.03	0.015
Repetition Time [s]	2	2	2.5	2	2
Pixel spacing [mm]	3x3.1	3x3	2.5x2.56	3x3x4	3x3x3
Slice thickness [mm]	3	2.5	2.7	3	4
Orientation	j-	j-	j-	i	i
Flip Angle [deg]	90	90	90	82	90
Duration [min]	07:06	05:14	07:00	06:00	06:00
T1					
Repetition Time [s]	0.0084	2.53	0.0094	0.00325	0.00325
Echo Time [s]	Shortest	0.0035	0.0046	0.00325	0.00325
Flip Angle [deg]	8	7	8	7	7

Site	OHSU_1	TCD_1	UCLA_1
number included	18	17	8
Manufacturer	Siemens	Philips	Siemens
Model	TriTim	Achieva	TriTim

Field Strength [T]	3	3	3
BOLD fMRI			
Echo Time [s]	0.03	0.027	0.028
Repetition Time [s]	2.5	2	3
Pixel spacing [mm]	3.8x3.8	3x3	3x3
Slice thickness [mm]	3.8	3.2	4
Orientation	j-	j-	j-
Flip Angle [deg]	90	90	90
Duration [min]	05:07	07:06	06:06
T1			
Repetition Time [s]	2.3	0.0084	2.3
Echo Time [s]	0.00358	0.0039	0.00286
Flip Angle [deg]	10	8	9

Subsample Characteristics

***Table S2**: Characteristics of the subsample without motion outliers (ASC: n=173, CMP: n=194). Abbreviations: ASC: autism spectrum condition, CMP: comparison group (no diagnosis), DVARS: D referring to temporal derivative of timecourses, VARS referring to RMS variance over voxels* FD: frame-wise displacement*, FIQ: full-scale IQ*

	mean	SD	min	max	p	d
Age [years]						
ASC	12.30	3.091	5.53	18.00		
CMP	12.70	2.871	6.36	18.00	0.202	-0.13
FIQ [scaled score]						
ASC	107.20	11.525	84.00	129.50		
CMP	110.29	10.416	84.00	129.00	0.008	-0.28
FD [mm]						
ASC	0.16	0.076	0.03	0.34		
CMP	0.13	0.067	0.04	0.36	<0.001	0.38
DVARS [%]						
ASC	2.92	0.627	1.35	4.03		
CMP	2.81	0.633	1.36	4.08	0.083	0.18

* calculated following to Power et al. 2011 *NeuroImage* doi:10.1016/j.neuroimage.2011.10.018.

Image quality metrics

The image quality metrics were calculated using MRIQC v0.14. The metrics were: AFNI's oulier ratio (aor), AFNI's image quality index (aqi), number of dummy TRs (dummy_trs), standard deviation of dummy TRs (dummy_std), entropy focus criterion (efc), foreground-background energy ratio (fber), framewise displacement mean (fdmean), number of timepoints above FD threshold [>0.2mm] (fd_num), smoothness average (fwhm), smoothness in x, y, or z-direction (fwhm_x, fwhm_y, fwhm_z), percentage of timepoints above FD threshold (fd_perc), global correlation (gcor), ghost-to-signal ratio in two directions (gsr_x, gsr_y), signal-to-noise ratio (snr), temporal signal-to-noise ratio (tsnr), background signal [mean, median, standard

deviation, median absolute deviation, 5%ile, 95%ile] (summary_bg_mean, summary_bg_mad, summary_bg_stdv, summary_bg_median, summary_bg_p05, summary_bg_p95), foreground signal [mean, median, standard deviation, median absolute deviation, 5%ile, 95%ile] (summary_fg_mean, summary_fg_mad, summary_fg_stdv, summary_fg_median, summary_fg_p05, summary_fg_p95).

See Table S3 for the Pearson correlation coefficients for all binary combinations of metrics. To reduce the dimensionality of the quality metrics, we conducted a principal component analysis (PCA) without factor rotation using the psych package for R (Revelle, W. (2019) psych: Procedures forPersonality and Psychological Research, Northwestern University, Evanston, Illinois, USA, https://CRAN.R-project.org/package=psych Version=1.9.12). The first factor explained the largest share of the variance (38%). The second, third, and fourth factor added 13, 12, and 10% of additional explained variance. Additional factors explained less than 10% of variance and were dropped. The 4-factor model was sufficient (empirical χ^2=1335.38, p=1.2e-123, RMSR: 0.06, total explained variance: 73%). The first factor loaded most strongly on background and foreground image intensity measures, the second factor loaded on smoothness measures, the third factor loaded on measures of displacement, and the fourth factor loaded on image quality and entropy measures (see Table S4).

Table S3: Correlation of image quality metrics

	1	2	3	4	5	6	7	8	9	10	11	12	13	14	15	16	17	18	19	20	21	22	23	24	25	26	27	28	29	30
1. aor																														
2. aqi	0.10																													
3. dummy_trs	-0.13	0.22																												
4. efc	-0.11	0.30	-0.09																											
5. fber	0.03	-0.06	-0.06	-0.19																										
6. fd_mean	0.58	0.42	0.00	-0.11	0.10																									
7. fd_num	0.25	0.46	0.05	-0.07	0.13	0.83																								
8. fd_perc	0.38	0.38	0.02	-0.12	0.11	0.91	0.90																							
9. fwhm_avg	-0.06	-0.18	-0.08	-0.36	0.06	0.04	0.20	0.04																						
10. fwhm_x	-0.11	-0.14	0.13	-0.29	0.09	0.03	0.16	0.04	0.88																					
11. fwhm_y	-0.05	-0.20	-0.01	-0.29	0.06	0.06	0.17	0.06	0.92	0.89																				
12. fwhm_z	0.01	-0.10	-0.30	-0.33	0.01	0.02	0.16	0.00	0.73	0.38	0.46																			
13. gcor	0.24	-0.08	-0.10	-0.15	0.05	0.18	0.12	0.13	0.09	0.05	0.07	0.10																		
14. gsr_x	-0.28	0.36	0.44	0.11	-0.07	-0.13	0.00	-0.13	0.12	0.28	0.08	-0.05	-0.04																	
15. gsr_y	0.18	-0.14	-0.37	0.47	-0.08	0.03	0.00	0.01	-0.11	-0.28	-0.09	0.07	-0.06	-0.64																
16. snr	-0.11	0.50	0.16	0.27	0.12	0.01	-0.01	0.03	-0.47	-0.29	-0.40	-0.49	-0.04	0.31	-0.37															
17. summary_bg_mad	-0.06	-0.04	-0.44	0.26	-0.11	-0.07	0.10	-0.08	0.50	0.24	0.35	0.66	0.04	-0.04	0.40	-0.29														
18. summary_bg_mean	-0.04	-0.08	-0.51	0.36	-0.09	-0.08	0.05	-0.10	0.39	0.13	0.27	0.57	0.01	-0.22	0.58	-0.30	0.92													
19. summary_bg_median	-0.02	0.09	-0.48	0.51	-0.13	-0.02	0.14	-0.04	0.21	-0.03	0.08	0.46	-0.02	-0.14	0.59	-0.25	0.88	0.89												
20. summary_bg_p05	0.12	0.04	-0.23	0.47	-0.10	0.03	-0.13	0.03	-0.59	-0.62	-0.53	-0.37	-0.10	-0.54	0.57	0.10	-0.15	0.12	0.16											
21. summary_bg_p95	-0.06	-0.17	-0.45	0.21	-0.04	-0.12	-0.01	-0.13	0.50	0.27	0.39	0.59	0.03	-0.20	0.47	-0.30	0.84	0.95	0.71	0.01										
22. summary_bg_stdv	-0.04	-0.22	-0.45	0.05	-0.01	-0.11	-0.01	-0.12	0.55	0.30	0.43	0.64	0.07	-0.24	0.39	-0.30	0.80	0.90	0.62	-0.03	0.97									
23. summary_fg_mad	0.00	-0.29	-0.42	-0.14	-0.02	-0.08	0.02	-0.08	0.66	0.37	0.50	0.78	0.12	-0.22	0.25	-0.43	0.78	0.79	0.54	-0.17	0.86	0.92								
24. summary_fg_mean	0.01	-0.26	-0.46	-0.09	0.00	-0.07	0.01	-0.08	0.58	0.31	0.44	0.70	0.14	-0.24	0.27	-0.29	0.77	0.81	0.55	-0.10	0.88	0.95	0.98							
25. summary_fg_median	0.01	-0.25	-0.46	-0.08	0.00	-0.07	0.01	-0.08	0.58	0.31	0.44	0.70	0.14	-0.24	0.27	-0.28	0.78	0.81	0.55	-0.10	0.88	0.95	0.97	1.00						
26. summary_fg_n	-0.13	0.05	-0.10	0.05	0.03	-0.06	-0.06	-0.08	0.29	0.42	0.38	-0.04	-0.15	-0.03	0.03	0.01	-0.03	0.03	-0.03	-0.03	0.06	0.05	-0.02	-0.02	-0.02					
27. summary_fg_p05	0.01	-0.24	-0.48	-0.06	0.02	-0.06	0.01	-0.07	0.53	0.27	0.41	0.65	0.14	-0.28	0.29	-0.24	0.75	0.81	0.54	-0.05	0.87	0.94	0.95	0.99	0.99	0.00				
29. summary_fg_p95	0.01	-0.27	-0.44	-0.11	-0.01	-0.07	0.01	-0.08	0.60	0.33	0.46	0.72	0.13	-0.25	0.27	-0.33	0.77	0.80	0.53	-0.11	0.87	0.94	0.99	1.00	0.99	-0.02	0.98			
30. summary_fg_stdv	0.01	-0.29	-0.42	-0.13	-0.02	-0.07	0.02	-0.08	0.64	0.36	0.49	0.76	0.13	-0.23	0.26	-0.40	0.77	0.79	0.53	-0.15	0.87	0.93	1.00	0.98	0.98	-0.03	0.96	0.99		
31. tsnr	-0.12	-0.75	-0.16	-0.23	0.14	-0.38	-0.42	-0.32	-0.08	-0.08	0.00	-0.12	0.07	-0.44	0.14	-0.13	-0.15	-0.05	-0.25	0.15	0.06	0.13	0.13	0.16	0.16	-0.08	0.18	0.16	0.15	

Table S4: Factor loading of the image quality metrics. Abbreviations: PC: principal component, Proportion: Proportion of the variance explained, Cumulative Var: Cumulative proportion of the variance explained, Proportion Expl: Proportion of the explained variance; Cumulative Prop: Cumulative proportion of the explained variance.

	PC1	PC2	PC3	PC4
aor	0.01	-0.26	0.59	-0.24
aqi	-0.21	0.08	0.47	0.72
dummy_trs	-0.53	0.37	-0.02	0.12
efc	0.09	-0.42	-0.14	0.73
fber	-0.05	0.06	0.13	-0.26
fd_mean	-0.06	-0.05	0.96	-0.02
fd_num	0.05	0.13	0.89	0.13
fd_perc	-0.07	-0.02	0.93	-0.02
fwhm_avg	0.57	0.73	0.12	-0.21
fwhm_x	0.28	0.82	0.06	-0.16
fwhm_y	0.43	0.69	0.10	-0.24
fwhm_z	0.72	0.35	0.13	-0.13
gcor	0.09	0.00	0.24	-0.27
gsr_x	-0.27	0.64	-0.13	0.51
gsr_y	0.46	-0.66	0.04	0.06
snr	-0.39	-0.06	-0.03	0.42
summary_bg_mad	0.89	0.06	0.00	0.29
summary_bg_mean	0.94	-0.15	-0.03	0.25
summary_bg_median	0.75	-0.23	0.05	0.47
summary_bg_p05	-0.02	-0.85	-0.03	0.10
summary_bg_p95	0.95	-0.02	-0.08	0.10
summary_bg_stdv	0.96	0.03	-0.07	-0.04
summary_fg_mad	0.95	0.16	-0.02	-0.18
summary_fg_mean	0.95	0.08	-0.02	-0.15
summary_fg_median	0.95	0.08	-0.02	-0.15
summary_fg_n	0.03	0.26	-0.08	0.06
summary_fg_p05	0.94	0.03	-0.02	-0.15
summary_fg_p95	0.95	0.10	-0.02	-0.17
summary_fg_stdv	0.95	0.14	-0.02	-0.19
tsnr	0.06	-0.27	-0.45	-0.69
Proportion Var	0.38	0.13	0.12	0.10
Cumulative Var	0.38	0.51	0.63	0.73
Proportion Expl	0.52	0.19	0.16	0.13
Cumulative Prop	0.52	0.71	0.87	1.00

Considerations regarding standard space transformation

The accuracy of standard space transformation can be a concern in samples that span a large age range. There are several reasons for using the MNI template in the current analysis. First, the study used pre-processed data to ensure the replicability of findings. The pre-processing carried out by the Functional Connectome Project deemed the MNI template appropriate for the ABIDE and ABIDE-II databases which span a wide age range (>5 years - >60 years). Second, participant

age is less critical for the transformation to the common space template than head size. Adult head size is typically achieved by 5 years of age. If any difference in head size due to age remains, it is less than the inter-individual variation in head size (see figure below). The individual variation is accounted for by using a combination of affine and non-linear transformation between individual and standard space.

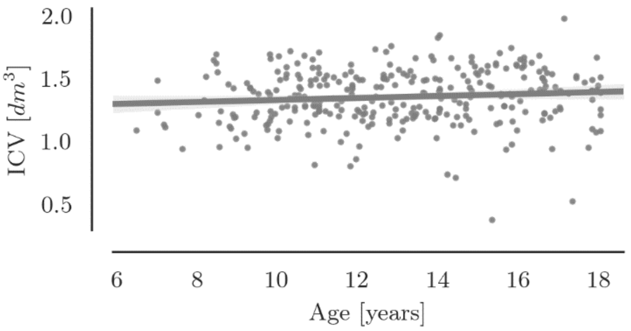

Figure S1 *Association between participant age and FreeSurfer-estimated intracranial volume (ICV) as a proxy for head size. There was no significant association between age and ICV in the sample.*

Considerations regarding ROI definition

The main reason for using 8-mm spherical ROIs is indeed to apply the same methodology as previous studies that investigated the subnetwork composition of the DMN (Andrews-Hanna et al. 2010; Andrews-Hanna et al. 2014). The alternative to using spherical ROIs would be to use anatomically-defined ROIs. However, the difference in size of the ROIs may introduce a confound in the connectivity analysis. For that reason, commonly used brain parcellations for functional connectivity analysis are either based on signal clustering to create roughly equal parcels, e.g. Craddock et al. 2011, Schaefer et al. 2018, or use spherical ROIs, e.g. Power et al. 2011. Because the hypotheses of the analysis present in the main manuscript concerned specific regions, we opted for the latter approach in keeping with the analyses by Andrews-Hanna and colleagues.
